# Recent advances of novel technologies for quality consistency assessment of natural herbal medicines and preparations

**DOI:** 10.1186/s13020-020-00335-9

**Published:** 2020-06-01

**Authors:** Xi-Chuan Wei, Bo Cao, Chuan-Hong Luo, Hao-Zhou Huang, Peng Tan, Xiao-Rong Xu, Run-Chun Xu, Ming Yang, Yi Zhang, Li Han, Ding-Kun Zhang

**Affiliations:** 1 School of Pharmacy, State Key Laboratory of Characteristic Chinese Drug Resources in Southwest China, Chengdu University of Traditional Chinese Medicine, No. 1066 Avenue. Liutai, Chengdu, 611137 China; 2 Sichuan Academy of Traditional Chinese Medicine, State Key Laboratory of Quality Evaluation of Traditional Chinese Medicine, Chengdu, 610041 China; 3 Jiangxi University of Traditional Chinese Medicine, Nanchang, 330004 China; 4 Chengdu Food and Drug Control, Chengdu, 610000 China

**Keywords:** Natural herbal medicines, Quality consistency assessment, Chemical evaluation, Biological evaluation, Chemical–biological integration evaluation

## Abstract

Quality consistency is one of the basic attributes of medicines, but it is also a difficult problem that natural medicines and their preparations must face. The complex chemical composition and comprehensive pharmacological action of natural medicines make it difficult to simply apply the commonly used evaluation methods in chemical drugs. It is thus urgent to explore the novel evaluation methods suitable for the characteristics of natural medicines. With the rapid development of analytical techniques and the deepening understanding of the quality of natural herbs, increasing numbers of researchers have proposed many new ideas and technologies. This review mainly focuses on the basic principles, technical characteristics and application examples of the chemical evaluation, biological evaluation methods and their combination in quality consistency evaluation of natural herbs. On the bases of chemical evaluation and clinical efficacy, new methods reflecting their pharmacodynamic mechanism and safety characteristics will be developed, and gradually towards accurate quality control, to achieve the goal of quality consistency. We hope that this manuscript can provide new ideas and technical references for the quality consistency of natural drugs and their preparations, thus better guarantee their clinical efficacy and safety, and better promote industrial development.

## Background

Natural herbal medicines are important complementary and alternative drugs in the world, which has played a crucial role in the development of human beings. At present, they are still the indispensable medical and health resources in China, Japan, Korea, India and other Asian countries. However, owing to differences in genetic backgrounds, geographical origins, planting environment, cultivation techniques, harvesting time, processing methods, exogenous impurities, and so on, the quality variation of natural medicines was significant [1], which is one of the main differences between natural medicines and chemical medicines.

Quality consistency is one of the basic attributes of medicines. Poor consistency makes it difficult to guarantee the effectiveness of drugs and increases the safety risk [2]. In the past, the quality consistency of natural herbs has not attracted much attention of clinicians, pharmaceutical companies and regulatory authorities. It was not until Dr. Weimar Shupei Pharmaceutical Factory in Germany used blending method to control the consistency of different batches of Ginkgo biloba leaves that pharmaceutical companies realized that the quality consistency of natural medicines with complex chemical composition could also be achieved [3]. Recent years, more and more attention has been paid to the quality consistency of natural herbs both in China and in other countries, especially in the process of industrial development [4]. For instance, Compound Danshen Dropping Pills, a modern Chinese medicine widely used in China for the treatment of cardiovascular diseases, were asked to provide measures to ensure product quality consistency between batches during the FDA registration process. In 2014, the FDA released “Guidance for Industry: Botanical Drug Products”. The guidelines state clearly that the most important point in the development of botanical drugs is ensuring that the quality and therapeutic effects of batches of drugs sold on the market are consistent [5, 6]. The latest guidelines issued by China Food and Drug Administration for the development of traditional medicines in 2018 also encourage the homogenization of medicinal materials to ensure the stability of the quality of preparations.

For the chemical medicines, the compositions are well defined, the quality consistency is generally determined by the measurement of in vitro dissolution curves, in vivo bioavailability [7, 8]. But for multi-component chemical drugs, especially natural herbs, the chemical composition is usually complex and the main active ingredients are not clear [9]. The main problems are mainly in the preparation process, composition analysis, quality research and so on. Under these conditions, how can we evaluate the quality consistency scientifically? The FDA guidelines point out that for drugs that cannot be fully evaluated by chemical methods, biological evaluation methods can be used. Among these ones, two botanical drugs have been approved for marketing, of which one is the green tea extract Veregen and the other is the resin extract Mytesi from the South American croton or dragon’s blood tree.

**Fig. 1:**
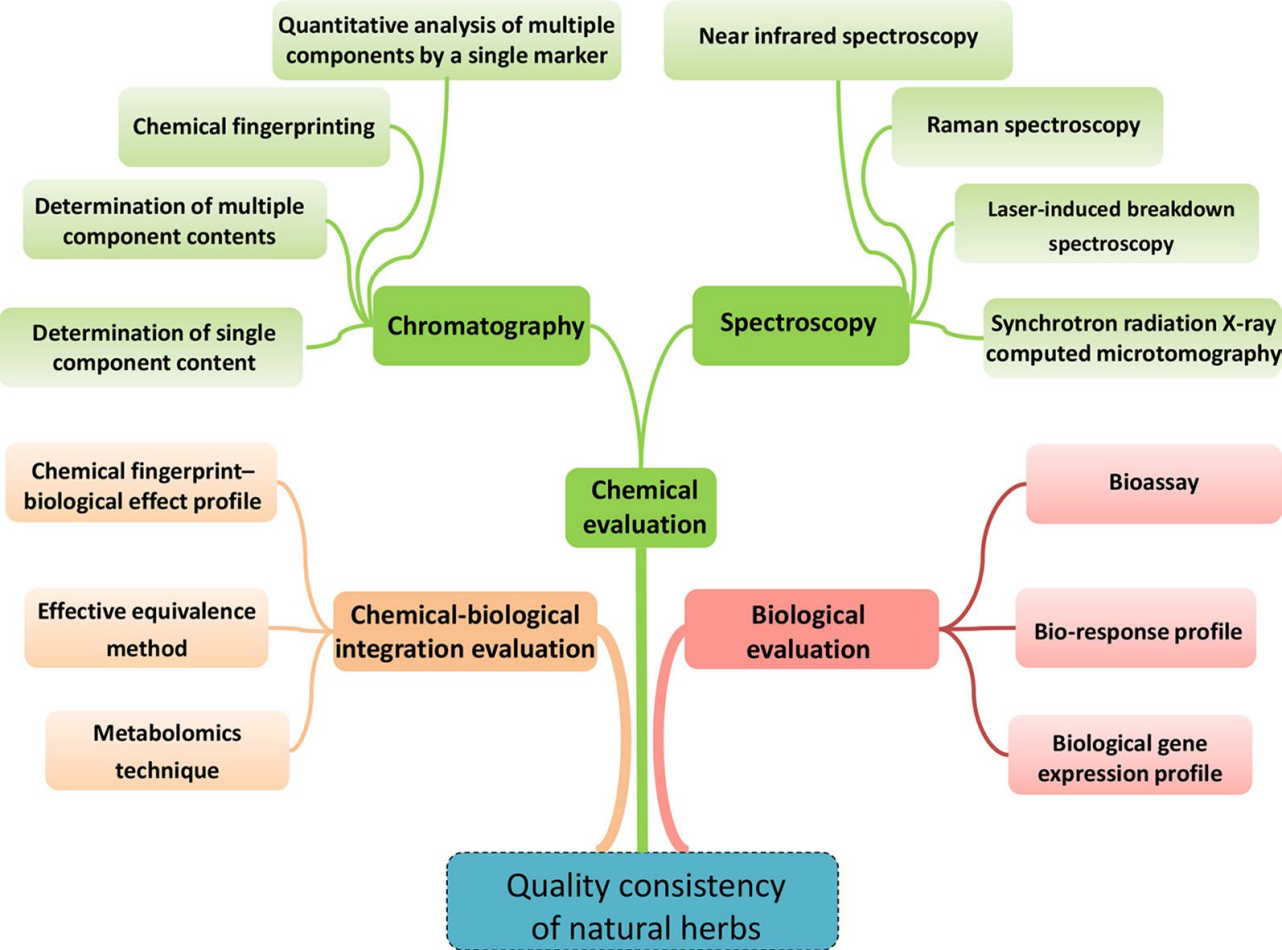
Overview for assessment methods of quality consistency

In view of the emergence of large number of methods for evaluating the quality consistency of natural herbal medicines in recent years, this manuscript mainly reviewed chemical methods, biological methods and their combination (Fig. 1). We hope that it can help to insight the nature of the quality of natural herbal medicine, explore scientific evaluation methods of quality consistency, and lay a foundation for the industrialization and clinical application of natural herbal medicine.

## Evolution of the concept of quality consistency assessment for natural medicines

The quality of natural herbs is fluctuating. In the age of lack of medical care, the volatility of quality does not affect the clinical treatment of herbal medicine to a large extent. After all, the first thing that needs to be protected is the accessibility of drugs [10]. However, with the great enrichment of social materials and the rapid improvement of medical standards, the quality fluctuation of herbal medicines has become a defect that cannot be ignored. The stable and controllable quality is the basic attribute of the drug. The excessive quality fluctuation makes the efficacy of the herbal medicine difficult to evaluate effectively, and even causes safety risks. The industrialized products produced are difficult to stabilize.

The concept of quality consistency evaluation of natural herbal medicines is to guide the development of conformity assessment methods to achieve chemical homogeneity or bioequivalence of natural herbs within a certain range. The evolutionary development process of the method for assessing the quality consistency of natural herbal medicines is essentially a process of deepening the understanding of the quality attributes of natural herbs and the scientific connotation of treatment [11].

Initially, the quality consistency assessment method of natural herbs is based on the quality control concept of chemical drugs, starting from the consistency of the content of a single component. With the deepening of the research on the effective substances of natural herbs, the characteristics of the multi-component interaction of natural herbs have been found, and the object of chemical consistency evaluation has also evolved from a single component to a multi-component content and the similarity or characteristic profile of the overall fingerprint. In fact, there is a certain deficiency in the consistency of multi-component content or the similarity of the overall fingerprint. The defect of consistency of multi-component content is that the actual operation process can hardly guarantee the complete consistency of each indicator component of different batches of natural medicine, unless a single target compound is artificially added without calculating the cost. Some studies have shown significant chemical typing of natural medicines from different sources. For example, rhubarb from different habitats has rhein and rhubarb phenolic forms [12]. At present, fingerprint similarity evaluation is considered as a relatively complete method, but its shortcomings are obvious. First, some components do not have UV absorption, such as polysaccharides, proteins, etc.; Second, the similarity of fingerprints is mainly determined by the high abundance peaks in the chromatogram, which weakens the contribution of some high-activity, low-content chromatographic peaks [13]. For example, the antiviral active ingredient (R,S)-Goitrin in the Radix isatidis is only about 0.02%, which is much lower than the content of adenosine and other components [14]. The fluctuation of the content of (R,S)-Goitrin can hardly affect the similarity evaluation results of the fingerprint. Combining the advantages and disadvantages of these two methods, the consistency evaluation method of fingerprint qualitative and active component quantitative double check is further formed, which not only monitors the overall contour but also individual components.

However, at the practical application level, various operability, time limit, and data analysis requirements have driven a new batch of technologies. Initially, in order to achieve the consistency evaluation under the condition of lack of standard materials, quantitative analysis of multiple components by a single marker (QAMS) was developed [15].

Due to the slow speed and low throughput of traditional chromatographic and mass spectrometry, it is impossible to monitor online. Chemical consistency evaluation gradually adopts technologies with fast, high efficiency and non-destructive advantages, such as Near-infrared spectroscopy (NIRS), Raman spectroscopy and Laser-induced breakdown spectroscopy (LIBS) and so on [16–18]. Especially, NIRS has played an important role in the online monitoring and consistency assurance of herbal industrial products, achieving a more general industrial application. In order to solve the cumbersome characteristics of chemical analysis, considering the overall characteristics of natural herbal medicines, some physical parameters, such as surface tension, pH, conductivity, viscosity, etc. [19]. It is also used for the consistency evaluation of herbal medicines by establishing a correlation model between physical parameters and chemical composition. This model is used in the industrial extraction of herbal medicines because these parameters are extremely easy to measure, analyze quickly, and regulate in time. Owing to the tremendous advances in chemical analysis, chemical evaluations often yield large amounts of even large amounts of chemical information. The data of consistency evaluation needs to be combined with principal component analysis, cluster analysis, partial least squares discriminant analysis and other chemometric methods to reduce the dimensionality, visually reflect the difference in quality consistency, and identify the key impact factors.

However, pharmacological activity is the core value of natural herbs. Although the chemical consistency-based quality consistency assessment method has made great progress, it still cannot answer the biological consistency. Especially, does the chemical consistency information obtained under specific conditions represent the consistency of biological effects; whether the volatility of chemical information indicates an inconsistency in biological effects. In fact, natural herbs are a complex system of chemical composition, and the dose–effect relationship is not clear or exists only in a specific concentration range. As a result, the concept of assessing the consistency of the activity of natural herbs through bioassays has received increasing attention. Bioassays are essentially quantitative pharmacology, which determines that test results are highly correlated with selected test models. The detection model can be human, whole animal, ex vivo tissues and organs, cells, organelles, bacteria, and even enzymatic reactions in vitro [11]. Generally, the more the test is completed at the overall level, the closer the result is to the actual efficacy of the drug, the less false positives, but the weaker the operability, the more difficult the data processing. At the same time, bioassays for consistency assessment must meet the technical requirements of quantitative analysis. From the aspects of method accessibility, analysis accuracy, speed, etc., it is a better choice to select carriers such as cells, bacteria or in vitro methods.

In addition to bioassays, bio-response profiles, biological gene expression profiles, biomarkers, etc. have been developed, taking into the complexities of biological reactions, aging changes, and dose–effect changes. In order to solve the flux problem of bioassay and increase the analysis rate, technologies such as biochips have been developed.

In order to solve the problem of the overall contribution of multi-component activity and content in chemical evaluation, some scholars have proposed the concept of effect component index, which is essentially a method for determining multi-component content based on activity correction [20, 21]. The technical advantages of high chemical precision, good popularity and bioassay associated activity are comprehensively utilized. On this basis, in order to give the actual pharmacological significance of the data, to facilitate the consistency guarantee operation such as blending batches, and to draw on the concept of explosive equivalent, the overall activity results of multiple components are converted into specific equivalent data of a single standard substance [22]. In addition, in response to the consistency assessment of toxic herbs, injections, etc., a combination of various chemical evaluation methods and biological evaluation methods has been successively derived.

From above, natural herbs have formed a multi-evaluation model with qualitative consistency of chemical evaluation, biological evaluation and its combined technology. For specific herbs or preparations, scientific quality conformity assessment methods should be developed based on their characteristics.

## Chemical consistency evaluation method

In 1985, the Chinese Pharmacopoeia began to compile methods for testing the content uniformity of chemical drugs and stipulated that the content uniformity of solid preparations should not vary by more than 15%. However, the confidence coefficients of two tests were 1.8 and 1.45, respectively, in the Chinese Pharmacopoeia and 2.4 and 2.0, respectively, in the United States, Britain, and Japan, which indicated that the reliability of the test method was low [14, 23]. Therefore, in order to promote the modernization of natural herbal medicines, more credible methods of assessment should be adopted.

### Chromatographic evaluation techniques

At present, the main chemical methods used to determine the quality consistency of drugs are chromatographic, including gas chromatography (GC), liquid chromatography (LC) and mass spectrometry (MS). In recent years, ultrahigh-performance liquid chromatography (UPLC) has gradually become popular and has been used in the analysis of the chemical compositions of natural herbal medicines [24].

In the evaluation of the consistency of the quality of natural herbal medicines, chromatography can be combined with MS to qualitatively and quantitatively analyze the composition of natural herbal medicines. The difference in consistency can be visually compared by mass spectrometry imaging [25]. Further, through chemical analysis means, the statistical methods of chemometrics are established, which can more accurately reflect the differences in the composition of natural herbal medicines, and provide a reference for the consistency evaluation of natural herbal medicines.

#### Determination of single component contents

Single-component quality assessment is a method used to control the quality of natural herbal medicines by determining the content of one of their effective components. However, natural herbal medicinal preparations are different from chemical drugs. Most chemical drugs only have one component, and the determination of the content of this single component can accurately indicate the consistency of the quality of the drug [26, 27].

In the early days, natural herbs reflected their quality consistency by measuring the content of a single ingredient. However, the ingredients of natural herbal medicines are complex, and their effects are the results of the combined effects of various ingredients. Differences in the contents of natural herbal medicinal preparations and the proportions of the ingredients will affect the efficacy of such preparations in clinical applications. Therefore, the quality consistency standards for natural herbal medicinal preparations have been extended to include quantitative determination methods based on multiple components and indices.

#### Determination of multiple component contents

The determination of multiple component contents is a method for controlling the quality of natural herbal medicinal preparations by measuring the contents of multiple active ingredients of natural herbal medicines or preparations. Tang et al. [28] used an HPLC–MS/MS method to identify the appropriate solvent and extraction method, determined the contents of 14 toxic and effective components in 29 batches of aconite formula granules, and assessed the consistency of their quality by a variety of chemometric methods.

Compared with the determination of a single component, the multi-component measurement is based on the comparison of multiple target components selected by it, which highlights the overall concept and makes a more scientific evaluation of natural herbs and their preparations. With the increase in the number of natural herbal medicines, traditional methods of assessment based on single components will gradually be replaced by the determination of multiple active ingredients.

#### Chemical fingerprinting

Chemical fingerprinting is a feasible method of confirming the authenticity of natural herbal medicines and assessing the consistency of their quality. Via the analysis of known and unknown components, it can fully indicate the overall situation of the complex system of natural herbal medicines [29]. At present, there are many examples of the application of chromatographic fingerprint method to determine the consistency of quality, and usually combined with chemometric methods for analysis, comprehensively reflecting the overall quality of natural herbs [30, 31]. Wang et al. [32] compared four kinds of chromatographic fingerprints of 71 batches of Xin-Ke-Shu using similarity analysis combined with hierarchical cluster analysis and finally proposed an effective strategy for assessing the consistency of their quality.

In recent years, Sun et al. have proposed a multi-component quantitative fingerprint technology that combines multi-wavelength measurement and metrological analysis to prove and verify the consistency of the quality of natural Chinese herbal medicine [33–38].

Compared with multi-component measurement, chemical fingerprints more comprehensively reflect the overall substance of natural herbs. If the active ingredients of herbs are not clear, chromatographic fingerprints can be provided to prove the consistency of product quality. Combined with fingerprint pharmacodynamics, the quality of natural herbs can be truly combined with their efficacy, which helps to clarify their mechanism of action.

#### Quantitative analysis of multiple components by a single marker

Quantitative analysis of multiple components by a single marker (QAMS) achieves the simultaneous determination of many other components of natural herbal medicines using a simple and easily measured component. It not only optimizes the detection of drugs but also dispenses with the use of standard substances and is a feasible method for assessing the effectiveness and internal quality of natural herbal medicines [39]. Li et al. [40] used HPLC to establish the fingerprint of *Achyranthes bidentata* Blume and, in combination with QAMS, analyzed the similarity and hierarchical clustering and assessed the quality of 18 batches of *A. bidentata*. The results show that there is no significant difference in the results of multi-component quantitative analysis established by the single-marker method and the external standard method, which can effectively determine the consistency of drug quality.

In the absence of chemical reference materials, the combination of chemical fingerprints and QAMS can still accurately and reliably determine the content of multiple ingredients in natural medicines, and further assess the consistency of their quality.

**Fig. 2:**
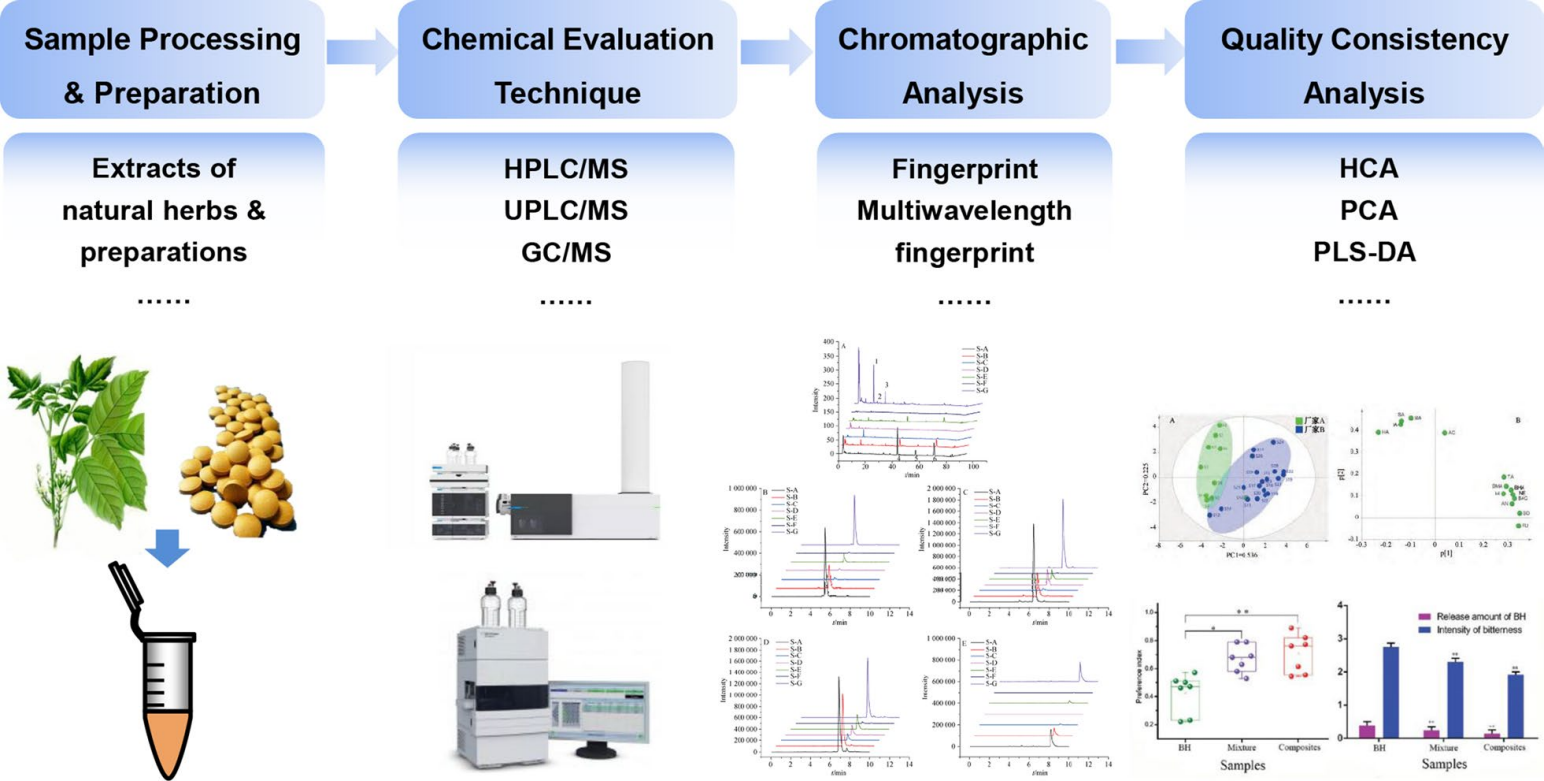
The flowchart of typical chemical assessment for consistency of quality

Chemical methods of assessment are still among the mainstream methods for assessing the consistency of quality (Fig. 2) (Table 1). Their advantages are that the methods are mature, the processes are simple, and the contrast that they provide is strong. However, their shortcomings are also obvious in that they require large number of preprocessing operations, which are time-consuming and laborious, and the use of reference substances is also extensive. The chromatographic evaluation method is mainly based on off-line detection methods, and is a detection method for production results. It cannot monitor the fluctuation of the quality index of natural herbs in the production process in real time, so it is impossible to control the quality consistency between batches of traditional Chinese medicine products. Therefore, in the process of producing pharmaceutical preparations in industry, to be able to control the quality of natural herbal medicines and their preparations online and in real time, methods for the assessment of the consistency of the quality of natural herbal medicines began to adopt spectral evaluation techniques.

**Table 1:** Quality consistency evaluation with chemical evaluation technology

Sample	Detection method	Analytical method	References
*Salvia miltiorrhiza* Bunge	Direct analysis in real time mass spectrometry (DART-MS)	PCA, Hotelling T(2) and dmodx	[24]
Hydrocortisone	HPLC–MS	Pharmacopoeial standards	[26]
Low-dose drug products	UPLC	Response surface methodology	[27]
Aconite formula granules	HPLC–MS/MS	CA, PCA, PLS-DA	[28]
*Melissa officinalis* L.	HPLC fingerprint	SA, PCA	[29]
Shenmai injection	HPLC fingerprint	PCA, Hotelling T(2) and dmodx	[30]
Shaoyao-Gancao decoction	LC–MS/MS, HPLC fingerprint	SA, HCA, PCA	[31]
Xin-Ke-Shu (XKS) tablets	Four types of chromatographic fingerprint	SA, HCA, Mahalanobis distance	[32]
Niuhuang Jiedu Pills	Integrated quantitative fingerprint	Wavelength Fusion Profiling, HCA, PCA	[34]
Compound Bismuth Aluminate tablets	HPLC multi-wavelength fingerprint	Simple quantified ratio fingerprint method, PLSR	[35]
Compound liquorice tablets	Multi-wavelength fusion HPLC fingerprints, UV fingerprint, UHPLC–ESI-Q-TOFMS	Averaged linear quantified fingerprint method, flow injection analysis	[36]
Liuwei Dihuang Pills	HPLC five-wavelength overall fused fingerprints, UV spectroscopic fingerprint	SA, PLSR	[37, 38]
*Achyranthes bidentata* Blume	HPLC fingerprint, QAMS	SA, HCA	[40]
Fufanggancao tablets	Quantitative HPLC fingerprint	SA	[41]
Ginkgo biloba leaves	UPLC–MS/MS	SA	[42]
Banxia Baizhu Tianma decoction	HPLC fingerprint	PCA, SA, information entropy	[43]
Kudiezi injection	HPLC–UV fingerprints, GC–MS fingerprints, HPIEC fingerprints	SA, HCA	[44]
Cicadae Periostracum	UPLC–QTOF–MS/MS	SA	[45]
Xuesaitong Dropping Pills	UPLC fingerprint	SA, PCA, OPLS-DA	[46]
*Desmodium styracifolium*	HPLC fingerprint	SQFM	[47]
Xiaojin Pills	HPLC–MS/MS	PCA, HCA	[48]
*Ganoderma lucidum*	HPTLC fingerprints, GC–MS fingerprints, PACE, HPSEC-meid	HCA	[49]
Yinqiaojiedu tablets	Three wavelength fusion HPLC fingerprint	SQFM, PCA	[50]
*Guava* leaf	HPLC–TOF-ESI–MS Fingerprint	HCA, PCA	[51]
*Sambucus nigra* L.	HPLC fingerprint	HCA, PCA	[52]
Hyangsapyeongwisan	UPLC fingerprint	SA	[53]
Liuwei Dihuang Pills	HPLC fingerprint	SA, PCA	[54]
Qingfu Guanjieshu capsules	HPLC fingerprint	SA	[55]
Yiqing preparations	HPLC fingerprint	SA	[56]

### Spectral evaluation techniques

Spectroscopic technology can quickly analyze the comprehensive properties of the samples, and design, control and analyze the production and processing process by measuring the key quality, quality and characteristics of the original medicinal materials and the materials in processing in real time. Similarly, the spectrum evaluation technology is like the chromatography technology. There are three levels in the development process: firstly, the single element content is analyzed by the spectrum technology, then it gradually develops into multi-element analysis, and finally it evolves into a comprehensive analysis of the overall elements of the sample. This article mainly briefly introduces the application of the following several spectral techniques in quality consistency evaluation.

#### Near-infrared spectroscopy

**Fig. 3:**
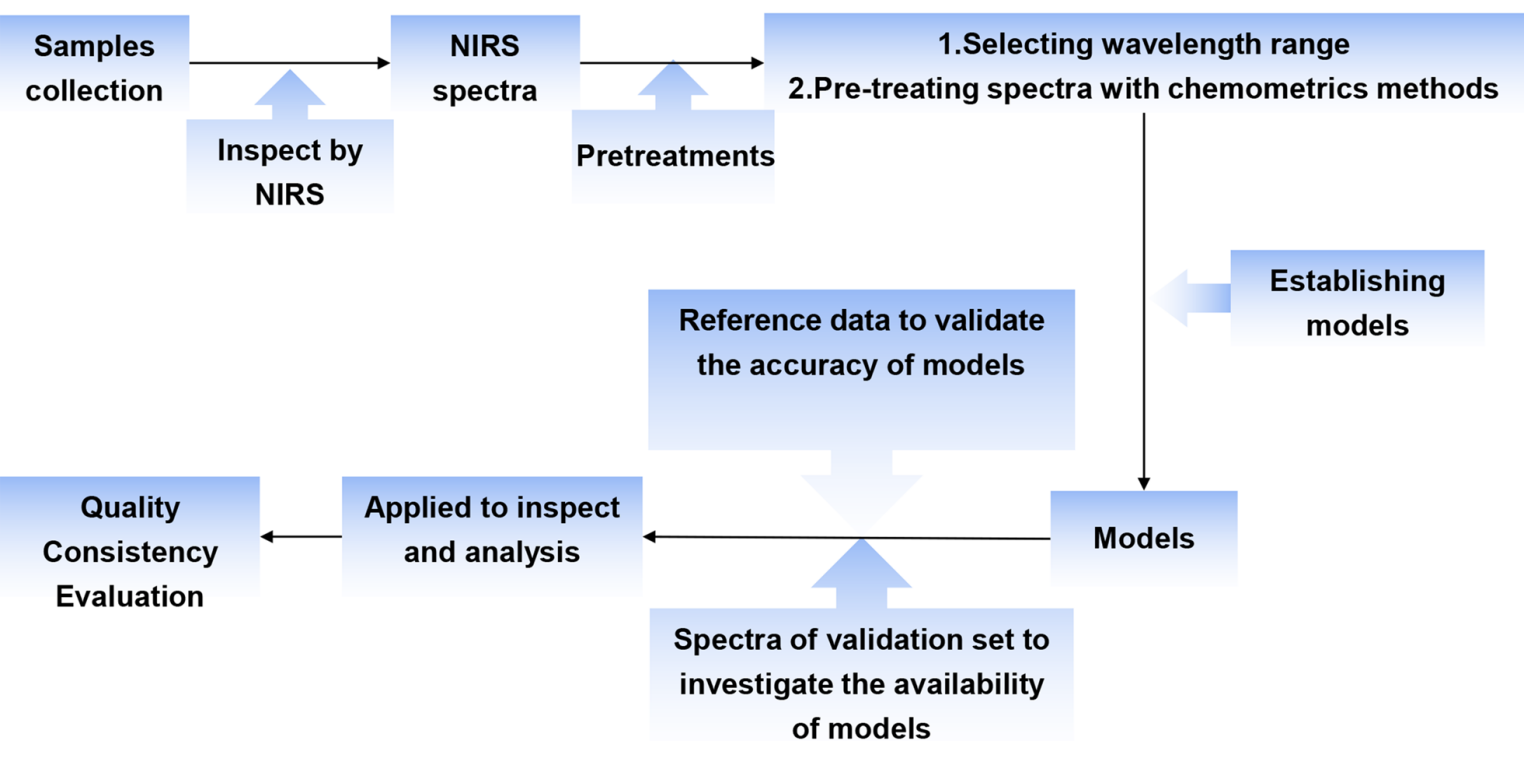
The basic process of NIRS for assessment of consistency of quality

Near-infrared (NIR) spectroscopy is an advanced green process analysis technique that relies on computer assistance to achieve rapid analysis and testing. In comparison with traditional analysis techniques, it has many advantages, such as high efficiency, rapidity, low energy consumption, and avoiding sample pretreatment, pollution, and destruction of the sample [57, 58]. The optical principles of NIR spectroscopy are mainly transmission spectroscopy and reflection spectroscopy. Characteristic information on hydrogen-containing groups in organic molecules in a sample can be obtained by analyzing the sample with an NIR spectrometer. A mathematical model of the sample can then be established using stoichiometry software, so that the relevant information can be obtained quickly [59–61]. In recent years, NIR spectroscopy has become popular and has been applied in many fields such as the petroleum sector, agriculture, industry, and medicine. It has also been applied in the field of natural herbal medicines [62–64]. Much research has shown that NIR spectroscopy has a wide range of application and can directly and quantitatively analyze original and compound natural herbal medicines and natural herbal medicinal preparations. Moreover, fiber optic probes can be used to achieve comprehensive continuous online monitoring of the production processes of natural herbal medicines [65, 66]. NIR spectroscopy can effectively enable the control of the consistency of the quality of natural herbal medicines (Fig. 3). Yang et al. [67] used NIR spectroscopy combined with multivariate statistical process control techniques, such as principal component analysis (PCA) or partial least-squares analysis, to study changes in quality during the extraction of honeysuckle extract from multiple batches. NIRS technology can monitor the changes of natural drugs in the production process online in real time, and then establish a model through chemometrics to analyze the spectral data, which is of great significance for improving the quality control level of modern natural herbal production [68, 69].

Near-infrared chemical imaging (NIR-CI) is an extension of NIR spectroscopy. By combining traditional visualization imaging with NIR spectroscopy, simultaneously analyzing the spectral and spatial distributions of a sample, visualizing its components, and finally forming a visualized image, it is an effective method for assessing the consistency of the quality of natural herbal medicines and their preparations [70–73]. Wang et al. [74] studied the relationship between the hardness and uniformity of different dispersible Yinhuang tablets using NIR-CI and used stoichiometry to measure the spatial uniformity of the tablet components. Finally, by the quantitative analysis of agglomerates of polyvinylpolypyrrolidone, the law governing the hardness and uniformity of the spatial distribution of dispersible Yinhuang tablets was revealed. Zhang et al. [75] adopted NIR DRS, established a mathematical model after first-order derivative and multiplicative scattering correction treatment and compared differences in the content of berberine in the production process that represented different levels of compatibility, which provided a basis for online monitoring of the production of natural herbal medicines.

NIRS is currently a popular technique for determining the quality of natural herbal medicines and represents a fast and effective method for assessing consistency of quality. NIRS can be applied to all key links in the production process of natural herbs and their preparations, such as extraction, concentration, alcohol precipitation, mixing, etc., to achieve quality control in the production process of drugs. As mentioned previously, it needs to be modeled in combination with chemometrics, but the model suffers from a certain amount of one-sidedness. Therefore, a focus of future research will be the analysis of spectra and direct examination of the resulting data [76]. Similarly, the current NIRS technology still has limitations such as high detection limit and unstable response value. The solution of these problems will accelerate the application of NIRS technology in the actual production process of natural medicine.

#### Raman spectroscopy

Raman spectroscopy is a fast and lossless technique for process analysis that can provide information on the frequency and intensity of molecular vibrations and can thus be used to analyze the structures and properties of substances. In recent decades, Raman spectroscopy has been widely used in the fields of polymers, pharmaceuticals, biological treatment, and biomedical analysis [77]. To some extent, Raman and NIR spectroscopy can complement each other in the analysis of molecular structures. Infrared spectroscopy records the transmission spectra of samples, and its principle is the change in the dipole moment; Raman spectroscopy records the emission spectra of samples, and its mechanism lies in the change in molecular polarizability. Both techniques can be used for the analysis of organic compounds, but for inorganic substances, especially oxides, it is difficult to obtain a complete spectrum by infrared spectroscopy, and the spectral bands are very wide, whereas the Raman spectral bands are very sharp. The effect of Raman spectral scattering is weak, and enhancement methods are often used in practical applications, such as surface-enhanced Raman spectroscopy, high-temperature Raman spectroscopy, resonance Raman spectroscopy, confocal Raman microscopy, Fourier transform Raman spectroscopy, and combinations with other techniques [78]. Modern portable and handheld Raman spectrometers are widely used in drug quality screening to detect low levels of substances or in combination with appropriate stoichiometric methods to distinguish compounds with similar structures and minimal spectral differences [79–82].

Raman spectroscopy has been widely used in the analysis of the compositions of pharmaceutical preparations and has been applied in the determination of the quality of mineral drugs in natural herbal medicine [83]. However, at present, the main application range of Raman spectroscopy is rapid detection, detecting the difference between the presence or absence of samples. For quantitative analysis in the process of drug production, Raman spectroscopy also needs to develop a corresponding model in combination with chemometrics. It is feasible to use the online Raman spectroscopy analysis technology to detect the production process of natural drugs, but the application of Raman spectroscopy in this area is still relatively small. How to design a stable and effective model is a matter of concern to researchers.

#### Laser-induced breakdown spectroscopy

**Fig. 4:**
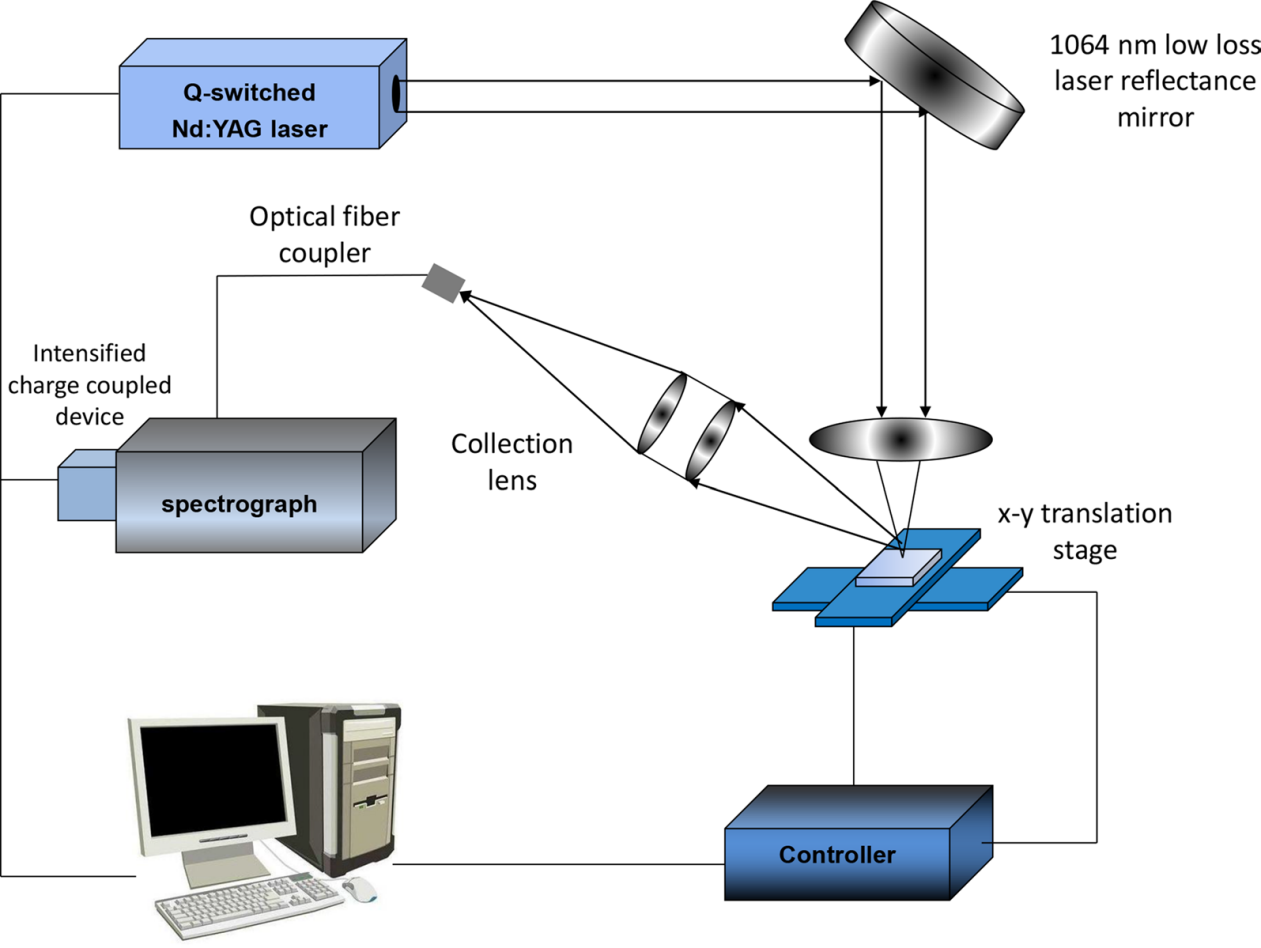
Schematic diagram of LIBS experimental device

Laser-induced breakdown spectroscopy (LIBS) is a form of atomic emission spectroscopy that involves the generation of a plasma using a laser as the excitation source. It is used for the rapid determination of the elemental contents and concentrations of materials by physical means (Fig. 4). Over the past two decades, LIBS has become a recognized and valuable analytical spectroscopic technique for the qualitative or quantitative analysis of the elemental properties of any type of sample [84]. With the development of optical technology, laser sampling and detection have gradually become mature. LIBS has been applied in industrial, agricultural, and pharmaceutical environmental monitoring, archeology, remote sensing, and other fields, as well in the quality control of natural herbal medicines [85]. By analyzing the distribution and quantitatively analyzing related elements in a drug, the consistency of the quality of the drug can be judged according to its elemental content [86]. Liu’s team has done a lot of research on LIBS technology in the rapid detection of natural drugs [87–91]. Wang et al. [92, 93] used LIBS to determine the contents of copper and lead in Rhizoma Chuanxiong and established a model in combination with multiple linear regression. Using LIBS in combination with PCA and an artificial neural network to analyze and identify the elemental compositions of Radix Angelicae Dangshen and *Polygonum cuspidatum* roots from different habitats and areas.

LIBS can be used to establish a method for the integral characterization of multiple elemental spectra in the quality control of natural herbal medicines. From the analysis of trace elements in natural herbal medicines in combination with chemometrics, visual data can be quickly obtained, and hence the consistency of the quality of different batches of natural herbal medicines and their preparations can be assessed [94]. At present, compared with near infrared spectroscopy and Raman spectroscopy, the sensitivity and accuracy of LIBS still need to be improved, but some characteristics of LIBS make it more suitable for elemental analysis. It is recommended to use double pulsed LIBS, magnetic confined LIBS and spark discharge LIBS to improve the sensitivity and accuracy of LIBS.

#### Synchrotron radiation X-ray computed microtomography

X-ray microtomography uses X-ray penetration to scan objects and then uses a computer for image processing to obtain images. It has been widely used for in vivo imaging of plants, insects, animals, and humans [95]. However, ordinary X-ray sources limit its development. Synchrotron radiation X-ray computed microtomography (SR-μCT) uses high-performance synchrotron radiation X-rays as a radiation source to collect three-dimensional structural information on samples, which has the advantages of high resolution, high discrimination, rapidity, and non-destructiveness, etc. This technique has unique advantages in the characterization of the structures of solid powders and particles [96, 97]. Li et al. [98] visualized the internal structures of sustained-release felodipine tablets in the process of drug release using SR-μCT, analyzed the surface morphology and changes in the structures of the tablets, and finally showed that SR-μCT is a new method for drug quality control. Wu et al. [99] determined the three-dimensional structure of the release hole in a captopril osmotic pump tablet by SR-μCT, observe its distribution clearly.

**Fig. 5:**
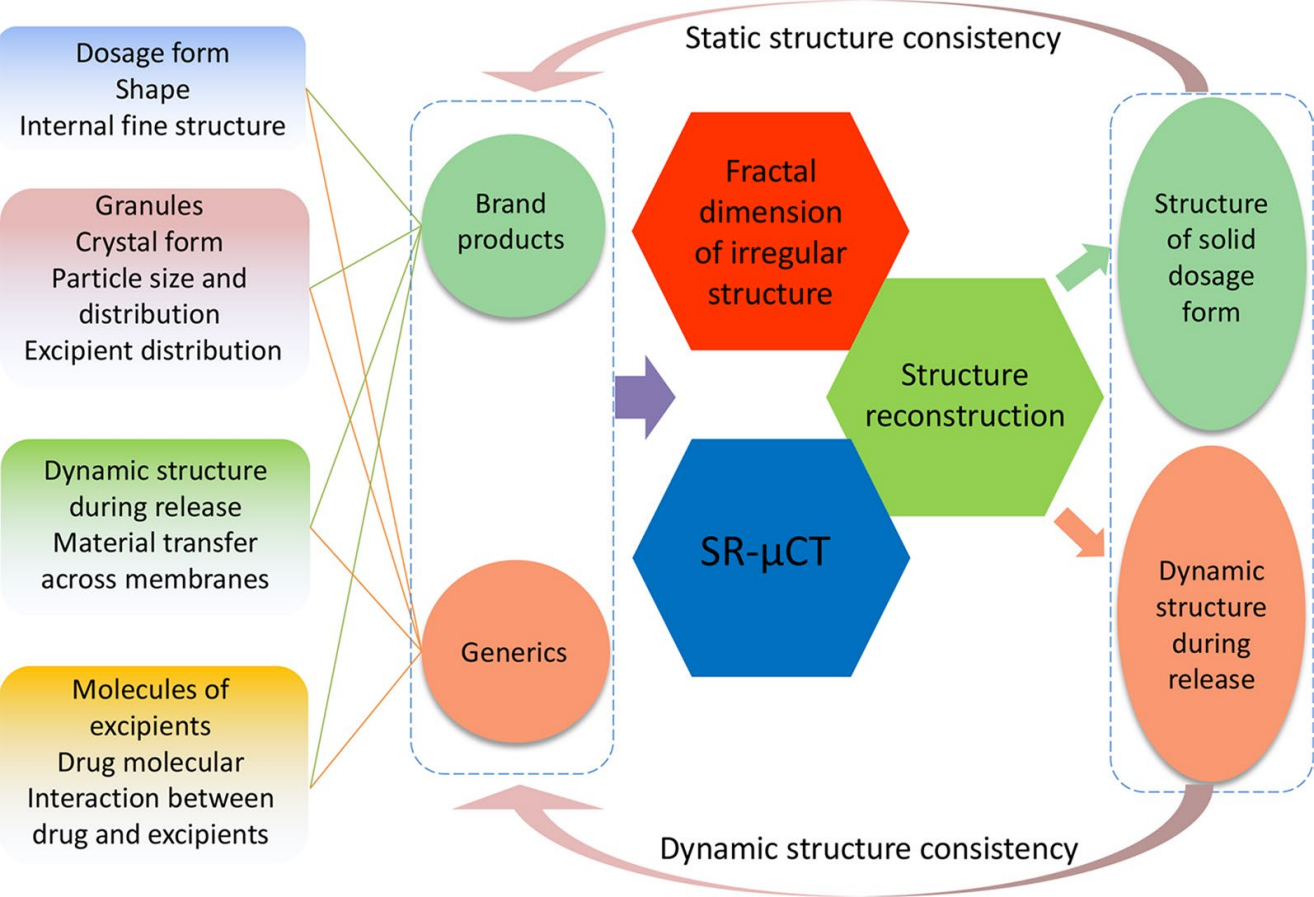
Road map for structure based strategy for consistency evaluation of dosage forms

When SR-μCT is used to study the three-dimensional structures and distributions of solid preparations, it can determine the sizes and distributions of particles in the body of the drug and enable the visualization and quantification of the structure of the preparation. Starting from the structure, it provides a new perspective on the assessment of the consistency of the quality of natural herbal medicines [100, 101] (Fig. 5). SR-μCT combined with 3D reconstruction, image processing, stereo modeling and quantitative analysis technology can realize the visualization and quantification of the preparation structure from both static and dynamic angles, and accurately quantify the fine structures of different levels. However, due to the requirements of the instrument, the detection object of SR-μCT is suitable for solid preparations and particle dispersion systems.

In addition to the above several new spectral techniques, there are some more commonly used spectroscopy techniques, such as ultraviolet spectroscopy, fluorescence spectroscopy and so on. The use of these technologies alone is no longer sufficient for current research needs, and is generally used in conjunction with new technologies to evaluate sample quality consistency [102–107]. Each spectral technique has outstanding advantages. However, in view of the current research status, the application of NIRS is higher than the other three spectral technologies from the perspectives of economy, practicality, and operability. Comparison on characteristics of four spectral techniques has been summarized in Table 2.

**Table 2:** Comparison of advantages of different spectroscopy techniques

	Accuracy	Quantitative	Reproducibility	Operability	Safety	Economy	Applicability
NIRS	+++	+++	+++	++++	++++	++++	+++++
Raman	++	++++	++	+++	+++++	++	+++
LIBS	+++	+++	++++	++++	++++	+++	++++
SR-μCT	++++	++++	+++	+++	++	+	++

**Fig. 6:**
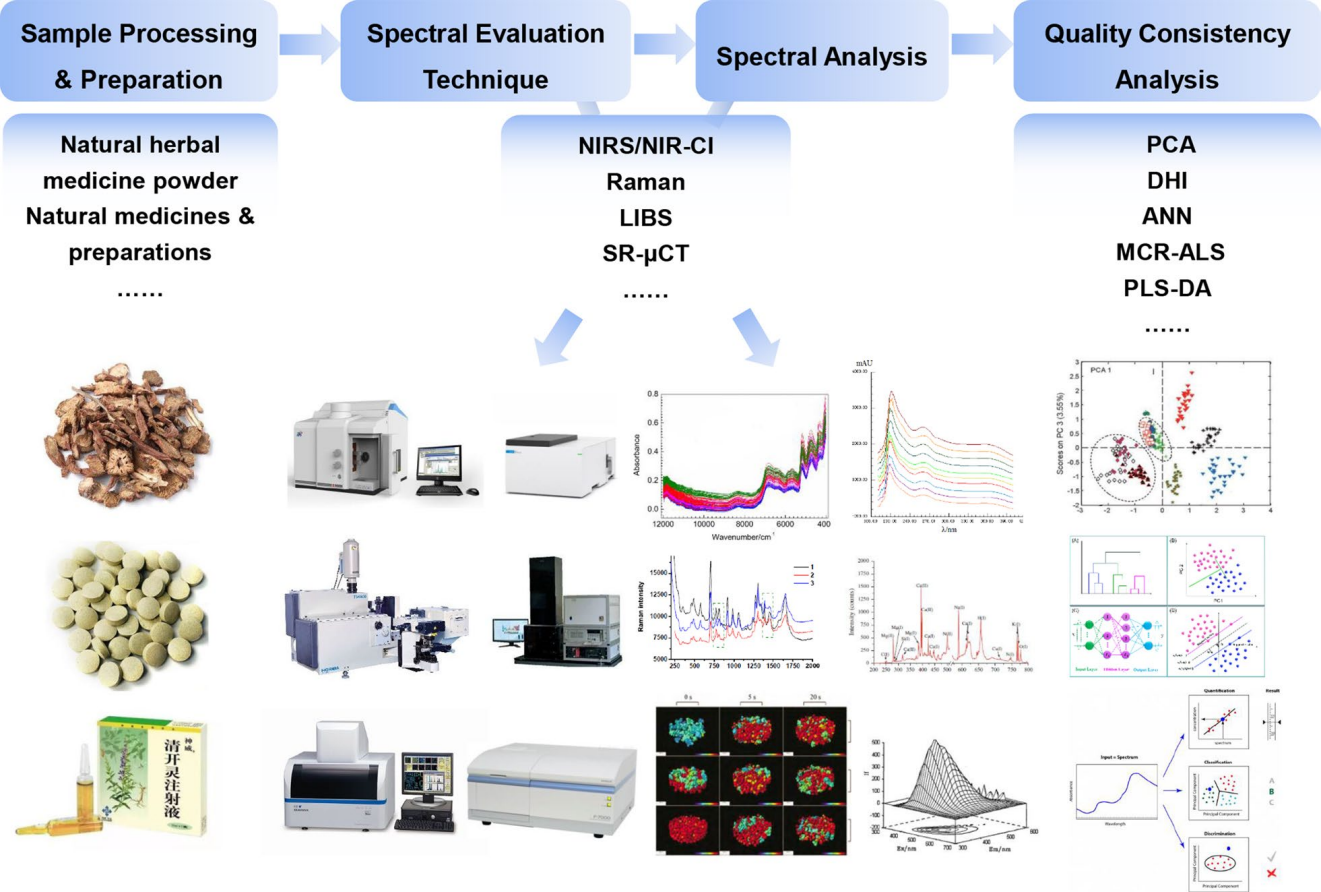
The flowchart of typical spectral assessment for consistency of quality

By the abovementioned spectral evaluation methods, the chemical consistency of the quality of natural herbal medicines can be determined and differences in consistency can be compared via the contents and distributions of the chemical components of such medicines (Fig. 6) (Table 3).

**Table 3:** Quality consistency evaluation with spectral evaluation technology

Sample	Detection method	Analytical method	References
*Lonicerae japonicae Flos*	NIRS	Multivariate statistical process control (MSPC) model, PCA	[67]
Cefazolin sodium pentahydrate Cephathiamidine	NIRS	One-way analysis, factor analysis, cluster analysis	[68]
*Ramulus cinnamomi*	Microscale thermophoresis On-line NIR-HPLC	Sipls, PLS model	[69]
Acetylsalicylic acid tablets	NIR-CI	Multivariate curve resolution-alternating least squares	[73]
Yinhuang dispersible tablets	NIR-CI	Basic analysis of correlation between analytes	[74]
*Coptis*	NIR diffuse reflectance spectroscopy	MSC, PLS, ANN	[75]
Rukuaixiao tablets	N/MIR diffuse reflectance spectral imaging	Correlation analysis method	[108]
Compound Liquorice tablets	NIR-CI	Basic analysis of correlation between analytes, PCA	[109]
*Flos Lonicera japonica*	NIR-CI	Basic analysis of correlation between analytes, moving block macropixel relative standard deviation	[110]
Shenzhiling oral Liquid	NIRS	PLSR, support vector machine regression (SVMR)	[111]
Wangbi tablets	NIRS	CARS-PLSR model	[112]
*Flos Lonicerae Japonicae*	NIRS	Synergy interval PLS (Si-GA-PLS)	[113]
Acetaminophen, SiO_2_, MCC, HPMC	Low-resolution Raman spectra	PCA, PLS	[79]
Entecavir tablets	Raman spectra, X-ray powder diffraction, differential scanning calorimetric	Thermo-gravimetric analysis (TGA)	[80]
Atropine sulfate	LC–MS/MS, Raman-CI, X-ray powder diffraction	SA	[82]
Cinnabar	Surface-enhanced Raman spectrum	Curve fitting	[83]
*Artemisia annua* L.	Laser Raman spectroscopy	Curve fitting	[114]
*Cornu Caprae Hircus*	Raman spectra,	PLS, HCA	[115]
Brufen, Glucosamine, Glucosamine, Paracetamol	LIBS	PCA, SIMCA	[86]
*Blumea balsamifera* DC	LIBS	PCA, PLS-DA model	[87]
Renqing Mangjue, Renqing Changjue, 25-herb coral Pills, 25-herb pearl Pills	LIBS	National Institute of Standard and Technology (NIST)	[88]
*Juncus effusus* L.	LIBS	Elemental microanalysis	[89]
An-Gong-Niu-Huang Wan	LIBS	RCCR, MWSD	[90, 91]
*Ligusticum wallichii*	LIBS	Multiple linear regression models	[92]
*Angelica pubescens* *Codonopsis pilosula* *Ligusticum wallichii*	LIBS	PCA, ANN, LDA	[93]
Sustained-release felodipine tablets	SR-μCT	Data visualization	[98]
Captopril osmotic pump tablets	SR-μCT	Data visualization	[99]
Advicor	First and third derivative UV spectrophotometry	H-point standard addition method	[102]
Qingkailing injection	UV spectra	LS-SVM	[103]
Propolis	NIRS, HPTLC, FLD	PLS-R, HCA, OPLS-DA	[107]
San-Huang tablets	Spectral quantitative fingerprinting (UV, FT-IR, CH)	SQFM	[116]

## Biological consistency evaluation methods

In recent years, the volume of natural herbal medicinal preparations exported from China has been much lower than that of natural herbal medicinal extracts. The main reasons are poor clinical efficacy and the incompleteness of the system for the assessment of bioavailability. Because a high-degree chemical similarity does not equal a bioequivalence. Therefore, the establishment of a complete system for the determination of bioavailability using biological techniques is the key factor in the assessment of consistency of quality and the modernization of natural herbal medicines [117, 118].

Biological assessment techniques are based on the biological effects of drugs. Experimental animals are used to obtain their tissues and organs for in vitro tests, microorganisms, cells, and related biological factors are used to determine the efficacy or toxicity of drugs, and thus the quality of drugs is assessed. Biological techniques are new methods for the quality control of natural herbal medicines in addition to chemical assessment of quality and have the advantage of overall controllability of pharmacodynamics [119]. Methods used for the biological assessment of the quality consistency of natural herbal medicines are different from general pharmacological experimental methods. Biological assessment methods for the study of the quality consistency of natural herbal medicines involve not only quantitative pharmacological details such as test design, quantitative indices, interval groupings, and reliability tests but also parameters of pharmaceutical analysis such as linear range, precision, and repeatability.

Biological assessment techniques have the advantage of determining correlative efficacy and are in principle applicable to the assessment of the quality consistency of all the natural herbal medicines. In particular for natural herbal medicines of which the complex contents cannot be determined by physical and chemical methods or the clinical biological activity cannot be measured, the biological assessment techniques introduced into the system for the quality control of natural herbal medicines can not only identify varieties and quality but also determine pharmacodynamics and even observe toxicity and side effects, further control of biological consistency.

### Bioassay

Bioassay is based on the efficacy of natural herbal medicines. Quantitative pharmacology and pharmacological analysis are used to characterize the biological effects of natural herbal medicines qualitatively and quantitatively to control or evaluate their internal quality. The techniques mainly comprise the biopotency assay, biological toxicity potency assay, and bioactivity limits assay, etc. [120].

Biopotency refers to the specific biological effects of the test and control substances on a biological system under specific test conditions, and bioavailability methods are used to titrate the intensity of the biological effect of the test substance against that of the reference substance. The biological titer used for determining toxicity is also known as the biological toxicity potency. Assays of biopotency (toxicity) are quantitative methods, and their results are easy to quantify and measure with consistency. Biopotency is mainly applicable to the assessment of natural herbal medicines of which the efficacy is unclear, the efficacy of genuine medicinal materials, the grades of pharmaceutical products and rare natural herbal medicines, trends in activity, and obvious dose–effect relationships within a certain dose range. Biological toxicity potency is mainly applied to the assessment of the quality of toxic natural herbal medicines and provides an objective and accurate technical guarantee for the quality control and rational use of such medicines. The bioactivity limits assay involves semiquantitative or qualitative methods that refer to specific biological effects (such as agglutination, death, or convulsions) on an experimental biological system when the dose of the sample reaches a specific value, and is a semi-quantitative or qualitative method. According to the specific experimental design scheme, methods for the determination of biopotency and biological toxicity can be divided into in vivo and in vitro assays.

Li et al. [121] established a method for the assessment of the quality of Radix Isatidis based on the measurement of its in vitro activity against influenza virus neuraminidase, which indicated that the inhibitory mechanisms of Radix Isatidis and oseltamivir phosphate may be identical. By the method of measuring the potency of the substance, namely, by the comparison of its dose–effect curve with that of the positive control drug oseltamivir, the method of biological assessment of the shape and phases of the curve can be used for the assessment of the quality of Radix Isatidis. Further explore the consistency of biological activity of Radix Isatidis. Han et al. [122] established a biological assessment method for determining the quality of Xuesaitong (XST) capsules that used an in vitro test of biological activity in the form of an anti-platelet aggregation bioassay, and the biological titer was determined by a parallel-line assay. The above provides a basis for the biological evaluation of natural Chinese herbal medicine preparations.

However, there are still few cases in which the assessment of the quality consistency of drugs is carried out using biopotency. This paper intends to quantitatively assess the quality of medicines via biopotency methods with reference to the abovementioned two examples to explore ideas for the development of appropriate biopotency methods in order to determine the consistency of the quality of natural herbal medicines and their preparations.

### Bio-response profile

Biological response profiles refer to a group of characteristic biological data or maps that represent the effects of the sample on a biological system under specific experimental conditions, usually with time–effect or dose–effect dependence.

The most widely used Bio-response profile is the microcalorimetry. Microcalorimetry is a new method for assessing drug quality based on thermodynamics. In one sense, the system of life on earth is a thermodynamic system, and the laws governing living organisms follow the laws of thermodynamics. Both the metabolism of organisms themselves and the interactions between drugs and the body are accompanied by energy conversion and thermal changes. Therefore, according to the abovementioned theories, the bioactivity of drugs can be determined by methods based on biothermodynamics. The changes in energy due to the interactions between different drugs and the body can be measured in real time online and efficiently, and the intrinsic quality of a natural herbal medicine is expressed as a characteristic “fingerprint,” namely, its bioactivity fingerprint. By combining certain thermodynamic parameters to establish a mathematical model, the efficacy of a drug can be determined. Wu et al. [123] used microcalorimetry to study the biothermographic fingerprint of the interaction between Qingkailing injection and *Staphylococcus aureus*. Via similarity analysis, the analysis of biothermodynamic parameters, and comprehensive cluster analysis, a new idea for the biological assessment of the consistency of the quality of Qingkailing injection was proposed. Sha et al. [124] used microcalorimetry to determine the effects of 11 batches of Lianhuaqing capsules (LQCs) on the growth and metabolism of *Pseudomonas aeruginosa* at different concentrations, which provided a methodological reference for the assessment of the consistency of the quality of other natural herbal medicinal compounds.

### Biological gene expression profile

With the rapid development of genetic biological information, the identification of organisms via genetic information is a popular research direction among researchers. The biological gene expression profile is a narrow expression of transcriptomics, which is a discipline that studies gene transcription and transcriptional regulation in cells at an overall level. The expression profile can reflect the full effect of the drug on the biological system, so this technology can be used to screen out the mRNA that can characterize the activity of natural herbs and to monitor and evaluate the consistency of biological activity between natural herbal batches. Sun et al. [125] used HepG2 human hepatoma cells as a biodetector and selected six batches of compound Danshen dripping pills. After treatment for 24 h according to four corresponding criteria, 10 mRNA sequences, which could represent the biological activity of drugs, were screened by a gene expression microarray and quantitative real-time polymerase chain reaction. These sequences corresponded to the *MMP1*, *CYP1A1*, *EPGN*, *RUNX2*, *C8orf4*, *OLR1*, *CLMP*, *AKR1C1*, *IL24*, and *APOL6* genes, respectively, which exhibit significant dose–effect dependence. Similarity analysis was carried out according to the differences in the expression levels of the indicator genes for each batch of drugs, and the consistency of the bioactivity of the compound Danshen dripping pills was further assessed.

Each of the above three biometric methods has its advantages. Bioassay is a relatively simple and convenient method, which is mainly used for the in vitro evaluation of natural herbs. The consistency of samples can be obtained by comparing with standard substances according to intuitive phenomena such as color or aggregation state. Biological response profiles can detect the dynamic changes of samples. In comparison with the bioassay, biological response profiles can provide more fingerprint information and indicate the dynamic influence of a drug on an organism. Different from the above two methods, the biological gene expression profile can continuously monitor the differences of samples through the detection of multiple genes, more accurately reflect the differences in the biological information of the samples, and further evaluate the differences in consistency. However, it has higher requirements for the precision and operability of the experiment, so it is suitable for further research and development.

### Other biological evaluation techniques

The research of biological evaluation technology is not only aimed at the study of drug bioequivalence, but more importantly, the quality of drugs is evaluated by the correlation of drug efficacy. The above biological evaluation methods have application examples to support. However, there are still many biological evaluation techniques without quality consistency application examples, which can be qualitatively identified and quantitatively evaluated, and have application prospects for quality consistency evaluation, such as biomarkers, high content analysis, biochips and so on [126–133].

Biomarker is an indicator that can be used to objectively measure and assess a pathological change in a normal biological process or the effect of a drug intervention, and it is an important indicator when an organism receives an intervention. Currently, biomarkers are mainly studied by genomics, proteomics, metabolomics, and other related technologies.

High content analysis is a technique in which cells are used as detection objects, a variety of different fluorescent labels are used, microscopic imaging is used to record images of cells in a multi-well plate, and information in the images is analyzed to detect intracellular material activity. High content analysis technology relies heavily on high-resolution cell imaging systems to enable rapid multivariate analysis of test drugs at the cellular or molecular level.

Biochip immobilizes large number of biomolecules such as nucleic acid fragments, polypeptide molecules, even tissue sections, cells, etc. on a chip (carrier) according to a set manner, and utilizes a specific affinity reaction between biomolecules to realize analysis of the ligand. It features fast, efficient, parallel processing and analytical automation.

**Fig. 7:**
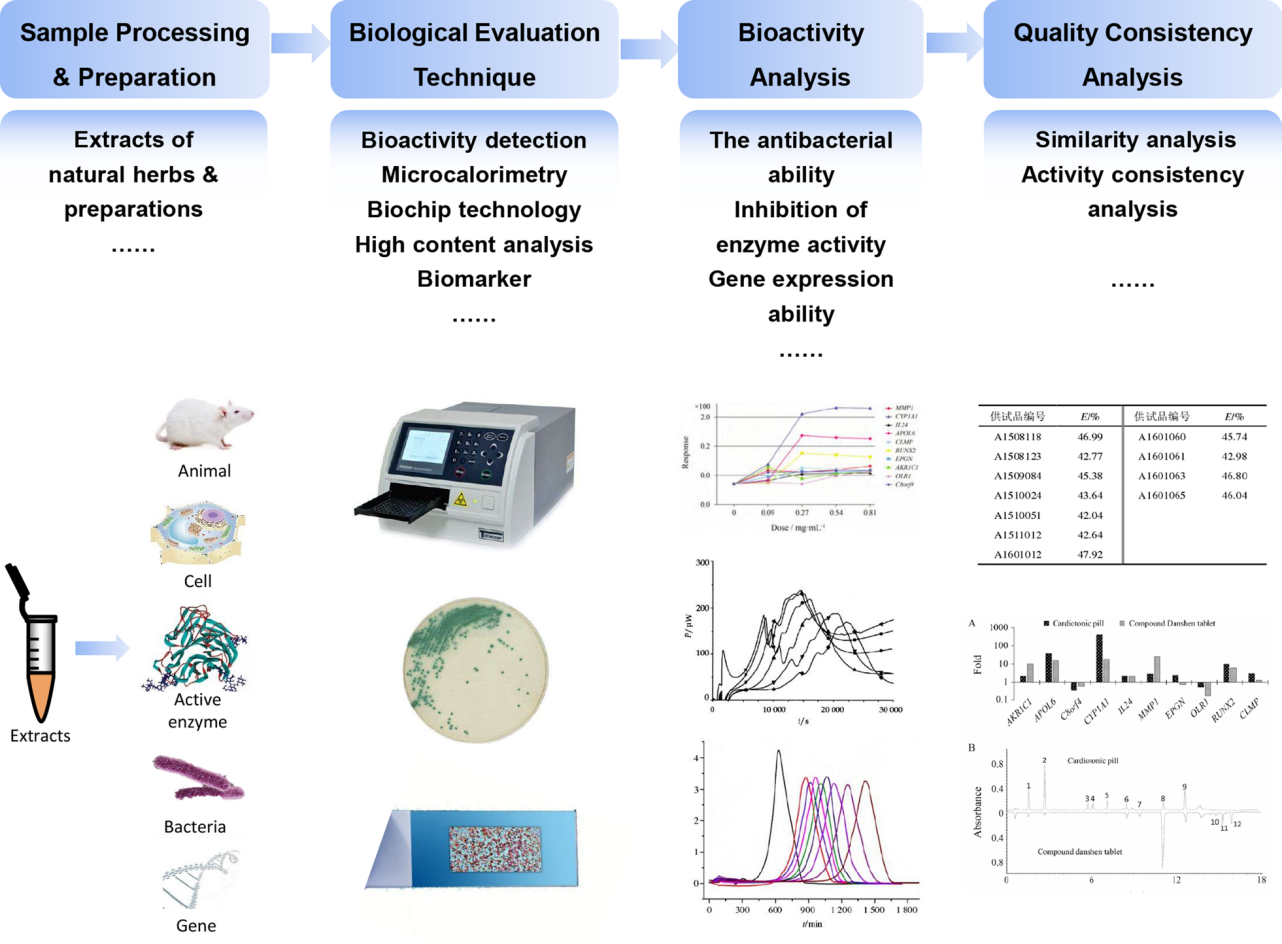
The flowchart of typical biological assessment for consistency of quality

The application of biological evaluation methods for quality control is directly related to the biological activity of natural herbal medicines and their preparations, and such methods can be used as a beneficial supplement to physical and chemical indicators and thereby more effectively and accurately determine the consistency and stability of the quality of natural herbal medicines (Fig. 7).

## Chemical–biological integration evaluation

Chemical–biological integration evaluation methods that can reasonably be deduced from the abovementioned chemical and biological assessment methods. Such methods not only use the chemical composition as a detection index but also examine the pharmacodynamics and mechanism of the active ingredients and avoid the shortcomings of quality control models based on “single-component theory.” It is difficult to determine the consistency of the quality of natural herbal medicines and their preparations if the assessment of quality is carried out using multiple indices alone without considering the different influences of different components on the overall efficacy of these medicines and preparations.

### Chemical fingerprint–biological effect profile

**Fig. 8:**
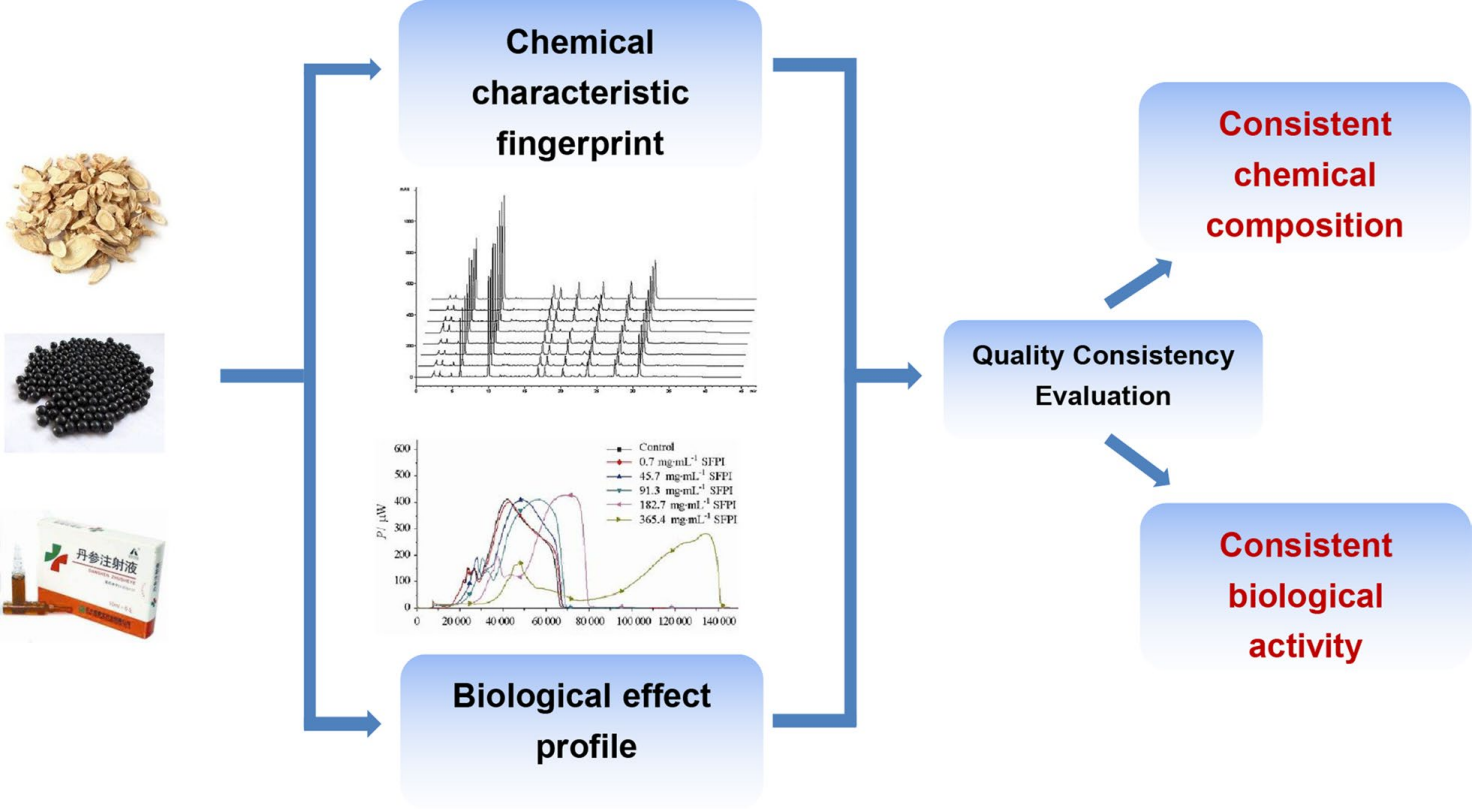
The flowchart of typical joint chemical–biological assessment for consistency of quality

Chemical fingerprinting can be used to characterize the chemical composition of a drug. To better illustrate the consistency of the quality of a drug, a microcalorimetric method can be used to generate a map of biothermal activity. The former determines the consistency of the quality of natural herbal medicines in terms of their chemical compositions, whereas the latter determines the consistency of their drug activity from the efficacy of their bioactive drug constituents and thereby control their quality more comprehensively (Fig. 8). Zhang et al. [134] established a method for the quality control of freeze-dried Shuanghuanglian powder for injection, which was based on mapping its chemical characteristics and biothermal activity. The map of chemical characteristics can effectively illustrate fluctuations in the contents of chemical components of the drug, and the map of biothermal activity can dynamically provide information on the overall biological activity of the drug, thereby assess the quality of freeze-dried Shuanghuanglian powder for injection more effectively. Cheng et al. [135, 136] proposed a general method for the quality control of plant medicines based on pharmacologically related chemical and biological fingerprints, namely, plant tissue quality control (PhytomicsQC), using LC/MS chemical characterization and fingerprinting, measurements of differential gene expression in cells for biological response fingerprinting, and in vivo pharmacological validation in animals. Eighteen batches of *Astragalus membranaceus* and pharmaceutical-grade samples of PHY906 were analyzed to assess the consistency of their quality. Large number of subsequent clinical studies found that PHY906 has the potential to improve an index of cancer treatment, whereas the effect of four batches of commercially produced baicalin decoction was not obvious. However, the abovementioned results cannot be obtained by LC/MS. Therefore, by employing 18 luciferase reporter cell lines and two enzyme assays based on the mechanism of PHY906, a mechanism-based quality control platform was established to distinguish between PHY906 and commercial *Scutellaria* extract.

Recently, researchers have proposed a new average linear quantitative fingerprinting method, combined with liquid phase analysis of antioxidant activity, successfully applied to the quality consistency evaluation of two herbal preparations. The relationship between the efficacy relationship of fingerprints and antioxidant activity was studied by orthogonal projection to latent structures method, which provided important pharmacodynamic information for quality control of traditional Chinese medicine [137, 138].

### Effective equivalence

**Fig. 9:**
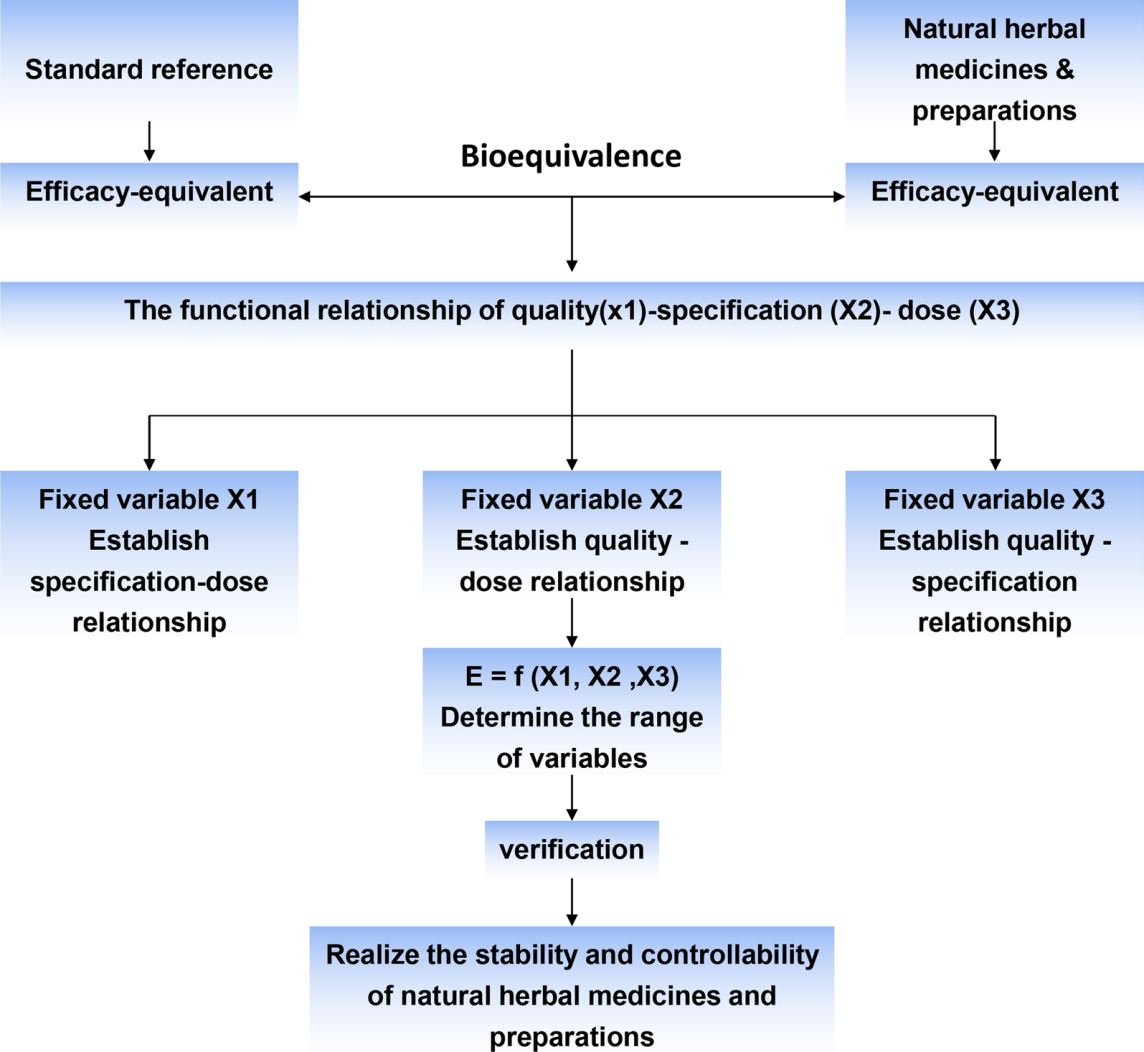
Research idea on the consistency of efficacy-equivalent [142]

Effective equivalent is a new concept of quality consistency evaluation of natural herbal medicines based on the total amount of biological effects exerted by effective substances. The effector equivalent is centered on the “effect-constituent index” [139], which is based on the common weighting of chemical composition analysis and biological effect detection (Fig. 9). The effect-constituent index calculates the sum of the effects of all pharmacodynamics or active ingredients based on the pharmacological effect or the biological activity intensity of the active ingredient as the weight of the chemical content, and usually reflects the quality information related to a specific efficacy of the natural herbal medicines. The establishment of the effective equivalence enables the quality standard of natural herbal medicines to achieve a practical correlation with the curative effect of the drug in the current controllable condition, and provides a reference for clinical application. The effective equivalence reflects the difference in quality between different medicinal materials. The high effect equivalent reflects the good quality of the medicinal material and the low effect equivalent, which reflects the poor quality of the medicinal material. This leads to the concept of “consistency of efficacy-equivalent,” which refers to the equivalence of the total biological effects of the active ingredients [22]. Its main function is to ensure that the pharmacodynamic effects of drugs produced in each batch are consistent by model biological calculations. In the course of clinical drug use, only the consistency of efficacy-equivalent is guaranteed, that is, the total amount of biological effects exerted by the effective substances is equal, so as to satisfy the quality, stability and consistency of the quality of traditional Chinese medicines to the greatest extent, and to ensure the safety and effectiveness of clinical drugs. Dong et al. [140] used the effective equivalence model to study the consistency of the dispensing of 18 samples of Rhizoma Coptidis with six different specifications by constructing an effect—constituent index of Rhizoma Coptidis and using PLS-DA, so as to further ensure the stability and consistency of the dispensing of natural herbal medicines in terms of their clinical efficacy. Similarly, Zhang et al. [141] took Rhizoma Coptidis as an example and proposed a comprehensive quality evaluation strategy for the consistency of traditional Chinese medicine formula granules with traditional decoctions.

### Metabolomics

The quality evaluation of natural herbal medicines based on metabolomics can more clearly clarify the complex components of natural herbal medicines and its pharmacodynamics and mechanism. Based on ^1^H-NMR metabolomics technology, the information of active ingredients of natural herbal medicines can be extracted and detected comprehensively, and the quality evaluation method that accords with the overall characteristics of the medicines can be established to ensure the consistency of the quality of the medicines [143–145]. Ma et al. [146] selected toad venom with anti-tumor activity as a model and developed a metabolite-based screening and quality consistency control (MSQCC) pipeline as a potential solution to this long-term problem. The study first demonstrated the efficacy of the metabolomics and biological profile correlation screening of 180 fractions prepared from natural heterogeneous venom samples to identify a series of bufadienolides as quality control markers for cancer cell inhibition. Finally, we developed a marker-based blending program (Markers-NMBT) to standardize the heterogeneity of natural medicinal substances (NMS). It creates blends that transform unqualified venoms with highly varying contents of bufadienolides, into qualified products that meet reference standards. Therefore, this work provides a strategie for rapid, large-scale discovery, quantification, and application of quality control markers to ensure consistency between batches and may be a key technology in the development of modern NMS formulations.

Chemical fingerprint–biological effect profile combines the chemical fingerprint and biological response spectrum to reflect the consistent changes in the chemical composition and biological efficacy of the drug. Its operation and application are relatively simple, and it can also observe the dynamic change process of the sample. Effective equivalence is to construct a mathematical model, express the chemical composition information and biological efficacy information with metrology, intuitively reflect the effective equivalent of the sample, and reflect the consistency of the drug. However, its application is still in its infancy, and there are not enough application examples. It needs to be further developed in the future. Metabolomics is currently a widely used and mature technology, but metabolomics has a large amount of information and needs to be compared with databases, so there are certain deficiencies in data visualization (Table 4).

**Table 4:** Quality consistency evaluation with biological evaluation and chemical–biological integration evaluation technology

Sample	Detection method	Analytical method	References
Scutellariae Radix	HPLC–PDA/QTOF–MS, antioxidant and anti-inflammatory bioactivities assays	Multivariate statistical analysis-based bridging, SA, HCA	[117]
Radix Isatidis	Neuraminidase activity assay	Parallel lines of qualitative effect	[121]
XST capsules	Antiplatelet aggregation	Parallel line assay	[122]
Xiaojin Pills	Antiplatelet aggregation	Simplified probit principle	[149]
Qingkailing injection	Bioactivity fingerprints	SA, One-way anova, CA	[123]
Lianhua Qingwen capsules	Microcalorimetry, growth metabolic thermogram	Regression analysis	[124]
Cardiotonic Pills	Gene chip, qrt-PCR, UPLC fingerprint	SA	[125]
Shuanghuanglian freeze-dried powder for injection	HPLC-ELSD fingerprints, Microcalorimetry	SA	[134]
Huangqin Tang, PHY906	LC/MS fingerprinting, bioresponse fingerprinting, in vivo validation, 18× Luciferase report cell lines	Phytomics Similarity Index (PSI)	[135, 136]
YIQING tablets	HPLC–DAD fingerprint, HPLC–DPPH assay	PCA, average linear quantitative fingerprint, multiwavelength total fusion profiling	[137]
Powdered Poppy capsules	HPLC fingerprint, Antioxidant Activities	Averagely linear-quantified fingerprint method, OPLS	[138]
Coptidis Rhizoma	HPLC fingerprint	Effective constituent equivalence, PLS-DA	[140]
Coptidis Rhizoma	HPLC, antibacterial zone	PCA, IC_50_	[141]
Huangqi injection solution	^1^H NMR Fingerprint	SA, relative content determination	[145]
*Bufo bufo gargarizans* Cantor	LC–MS/MS, Cytotoxicity assay in vitro, metabolomic	Markers-NMBT	[146]
*Salvia miltiorrhiza* Bunge	Micellar electrokinetic capillary chromatography fingerprint, antioxidant activity	SQFM, PCA	[147]
Tianma Toutong tablets	HPLC five-wavelength fingerprints, antioxidant activity	SQFM, PCA, PLS model	[148]
Lianqiao Baidu Pills	HPLC five-wavelength fingerprints, antioxidant activity	Limited-ratio quantified fingerprint method, PCA, PLS model	[150]
Sanhuang tablets	Capillary electrophoresis fingerprints, antioxidant activity	PLS model	[151]
Yinqiaojiedu tablets	Capillary electrophoresis fingerprints, antioxidant activity	PCA, PLS model	[152]
*Sophora flavescens*	Capillary electrophoresis fingerprints, antioxidant activity	Linear quantitative profiling method, PLS model	[153]
Weibizhi tablets	Micellar electrokinetic chromatography fingerprinting, antioxidant activity	Simple quantified ratio fingerprint method, PLS model	[154]
Fufang Danshen Pills	Micellar electrokinetic chromatography fingerprint, Antioxidant activity	SQFM, PLS model	[155]
*Ixeris sonchifolia*	Microemulsion electrokinetic chromatography fingerprints, antioxidant activity	PCA, OPLS	[156]
*Ixeris sonchifolia*	HPLC fingerprint, antioxidant activity	SQFM, SVM, PCA, PLS, OPLS	[157]
*Sophora* flower-bud, *Sophora* flower	HPLC-Q-TOF MS, antioxidant and hyaluronidase inhibitory activities	PLSR, BP-ANN, PCA	[158]
*Matricaria chamomilla* L.	HPLC fingerprint, antioxidant activity	PCA, HCA	[159]
Compound Danshen tablets	HPLC multiwavelength fusion fingerprints, antioxidant activity	SQFM, SA,	[160]
Lianqiao Baidu Pills	Micellar electrokinetic capillary chromatography fingerprint, antioxidant activity	Limited-ratio quantified fingerprint method, PCA, HCA, PLS model	[161]
*Isatidis Folium*	HPLC fingerprint, antioxidant activity	Equal weight quantified ratio fingerprint method, PLSR	[162]
Compound Liquorice tablets	HPLC multi-wavelength fusion fingerprint, antioxidant activity	Averagely linear quantified fingerprint method, HCA, PCA, PLS model	[163]
Zhenju Jiangya tablets	HPLC multi-wavelength fusion fingerprint, antioxidant activity	SQFM, PCA, PLS model	[164]

## Conclusion

The control of the quality consistency of natural herbal medicines has always been one of the key points and difficulties in their internationalization and modernization. With the modernization of natural herbal medicines, methods for assessing the consistency of the quality of natural herbal medicines have included advanced analytical techniques in the fields of chemical, physical, and biological assessment. Among the many evaluation methods mentioned in this article, they are applicable to the quality consistency evaluation of natural medicines. For scientific research, many scholars still use chromatography and further combine some biological activity evaluation for small-scale research. In industrial production, due to the concentration of research objects, more and more manufacturers have begun to use spectrometry to quickly and real-time check the consistency of pharmaceutical preparations. However, the clinical application of natural herbs has gradually deepened, and it is not appropriate to consider the consistency of the chemical composition alone, so the combination of chemical composition and biological activity for evaluation will be the future development trend.

For single herbals, the quality of a single herbs can be evaluated in both chemical and biological aspects; while multi-component herbal preparations should not only consider the effects of various herbal ingredients, but also consider how to control production online from the perspective of the formulation process to meet the consistency requirements of the formulation. In addition, it is necessary to consider the effect of the formulation dosage form on consistency, such as dissolution, degree of disintegration, and the like.

Modern methods for determining the consistency of the quality of drugs are more closely associated with their clinical mechanisms and have the advantages of rapidity, comprehensiveness, non-destructiveness, and efficiency. The mechanisms of action of drugs have been shown to be strongly correlated with their clinical effects. In particular, in the case of the natural medicinal system represented by natural herbal medicine, which involves complex components, we must not only monitor the components with high activity and high contents, that is, the iconic components, but also control the overall compositional profile and thus achieve the goal of “not only seeing the towering trees, but also the outline of the forest.”

This article has reviewed methods used for the assessment and control of the quality consistency in the complex system of natural medicine. We hope it can provide methodological references and guidance for the quality consistency evaluation, explore scientific evaluation methods of quality consistency, and lay a foundation for the industrialization and clinical application of natural herbal medicines, which will be benefit for the modernization and internationalization of natural herbal medicines.

## Data Availability

Not applicable.

## Abbreviations

LC: Liquid chromatography
GC: Gas chromatography
MS: Mass spectrometry
DART-MS: Direct analysis in real time mass spectrometry
QAMS: Quantitative analysis of multiple components by a single marker
NIRS: Near-infrared spectroscopy
LIBS: Laser-induced breakdown spectroscopy
SR-μCT: Synchrotron radiation X-ray computed microtomography
SQFM: Systematic quantified fingerprint method
SA: Similarity analysis
PCA: Principle component analysis
HCA: Hierarchical clustering analysis
PLS-DA: Partial least squares discriminant analysis
PLSR: Partial least squares regression
MSC: Multiplicative scatter correction
ANN: Artificial neural network
LDA: Linear discriminant analysis
FLD: Fluorescence detection
